# Evidences Suggesting that Distinct Immunological and Cellular Responses to Light Damage Distinguishes Juvenile and Adult Rat Retinas

**DOI:** 10.3390/ijms20112744

**Published:** 2019-06-04

**Authors:** Anna Polosa, Shasha Lv, Wassila Ait Igrine, Laura-Alexie Chevrolat, Hyba Bessaklia, Pierre Lachapelle

**Affiliations:** 1 Dept of Ophthalmology & Neurology-Neurosurgery, McGill University/Montreal Children’s Hospital Research Institute, Montréal, QC H4A 3J1, Canada; anna.polosa@mail.mcgill.ca (A.P.); shashalv0626@gmail.com (S.L.); wassila.ait@live.ca (W.A.I.); lauraalexie.chevrolat@gmail.com (L.-A.C.); amanemisa61@yahoo.fr (H.B.); 2 Health Science Center, Xi’an Jiaotong University, 76 West Yanta Road, Xi’an 710061, Shaanxi, China

**Keywords:** light-induced retinopathy, retinal structure and function, intrinsic resistance, immune response, neurotrophic factors, juvenile and adult rats

## Abstract

To unravel the mechanisms behind the higher resistance to light damage of juvenile (JR) versus adult (AR) rats, Sprague Dawley rats were exposed to a bright luminous environment of 10, 000 lux. The light-induced retinopathy (LIR) was assessed with histology, electroretinography and immunohistochemistry (IHC). In JR, 2 days of exposure induced the typical LIR, while >3 days added little LIR. IHC revealed a subtle migration of microglia (Iba1 marker) from the inner to the outer retina after 3 days of exposure in JR contrasting with the stronger reaction seen after 1 day in AR. Similarly, in JR, the Müller cells expressed less intense GFAP, CNTF and FGF2 staining compared to AR. Our results suggest that in JR the degree of retinal damage is not proportional to the duration of light exposure (i.e., dose-independent retinopathy), contrasting with the dose-dependent LIR reported in AR. The immature immune system in JR may explain the delayed and/or weaker inflammatory response compared to AR, a finding that would also point to the devastating contribution of the immune system in generating the LIR phenotype, a claim also advanced to explain the pathophysiology of other retinal degenerative disorders such as Age-related Macular Degeneration, Diabetic Retinopathy and Retinitis Pigmentosa.

## 1. Introduction

In albino rats, bright light exposure is known to trigger severe and irreversible retinal damage and there is growing evidence linking the pathophysiology of the rodent light-induced retinopathy (LIR) with human retinal disorders such as Retinitis Pigmentosa and Age-related Macular Degeneration [1,2]. The retinal damage is usually most pronounced in a well circumscribed region of the superior retina [3,4,5,6], a region referred by us as the “photoreceptor hole” (due to the fact that nearly all its photoreceptors are destroyed following light exposure). In contrast, damage in the inferior retina remains relatively uniform (i.e., no significant variations with eccentricity), at least in the early stages of the disease [6], thus creating distinctive superior-inferior hemiretinal differences which is the hallmark of the LIR model. Given that this photoreceptor hole (i.e., in the superior retina) is present immediately following the end of the light exposure, one aim of this study was to determine, in juvenile rats, the minimal duration of light-exposure required to cause the typical hemiretinal difference (i.e., first appearance of the photoreceptor hole in the superior hemiretina only).

Similarly, we have previously shown that compared to adult rats, juvenile rats are more resistant to LIR [4,6]. While in adult rats, a six-day long exposure to a luminous environment of 10,000 lux will almost completely destroy the photoreceptors, the retina of juvenile rats exposed for 14 days to the same environment will be significantly less affected [4,6]. The exact reason for this difference in the degree of photoreceptor damage between juvenile and adult LIR rats remains unknown. We showed that higher levels of neurotrophic factors (such as FGF-2 and CNTF) secreted by the Müller glial cells were expressed in younger compared to older light-exposed retinas [7]. Neurotrophic factors are known to play a significant role in photoreceptor survival in many models of retinal degeneration [8,9], implying that these factors could also constitute an important endogenous mechanism of resistance against light-induced damage in juvenile rats. Previous studies also suggested that the light-induced photoreceptor damage could be, in part, related to an inflammatory response from activated microglial cells [10,11]. It is believed that photoreceptor cell death triggers microglial migration towards the injured area to phagocytose and remove cellular debris and dying cells [12]. Although the primary role of these activated microglial cells is to defend the adjacent “healthy” retina against a potential ongoing oxidative stress (caused by dying photoreceptor cells), they also secrete different molecules (such as pro-inflammatory cytokines, reactive oxygen species, excitatory amino acids, etc. [13]) that can be harmful to surrounding healthy neurons. Based on the above, another aim of our study was to determine if age-dependent differences in the immune and cellular responses at the onset of the light exposure could explain the higher intrinsic resistance to light-induced damage found in juvenile rats compared to adult rats.

## 2. Results

### 2.1. Effect of Bright Light Exposure on the Retinal Function

**Figure 1:**
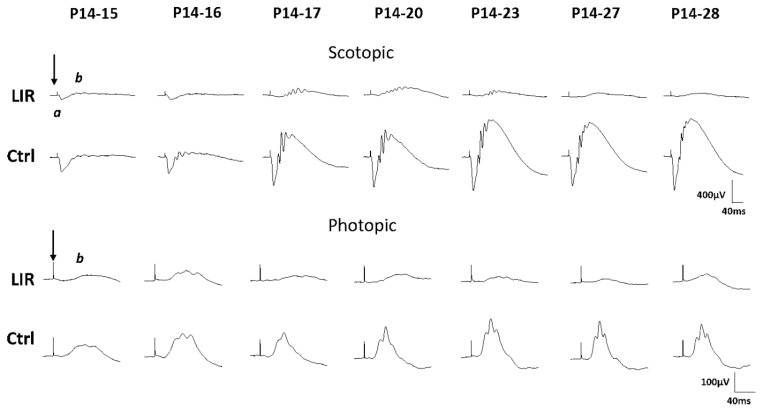
Representative scotopic and photopic ERGs recorded from light-exposed (10,000 lux) and control (unexposed) animals (*n* = 3–4 animals per group). For the exposed animals, all the ERGs were recorded immediately after the cessation of light exposure and compared to age-matched controls. For example, pups that were exposed for 1 day (P14–P15) were tested at P15 and compared to P15 controls. Horizontal calibration: 40 ms; vertical calibration: 400 µV (scotopic) and 100 µV (photopic). A 20 ms prestimulus baseline is included in all tracings. Vertical arrows indicate the flash onset. Abbreviations: a-wave (a) and b-wave (b). Light intensity used: 0.9 log cd.sec.m^−2^, flash duration 20 µs, inter stimulus interval 10ms (scotopic condition) and 1ms (photopic condition). A background light of 30cd/m^2^ was also used in photopic conditions.

**Figure 2:**
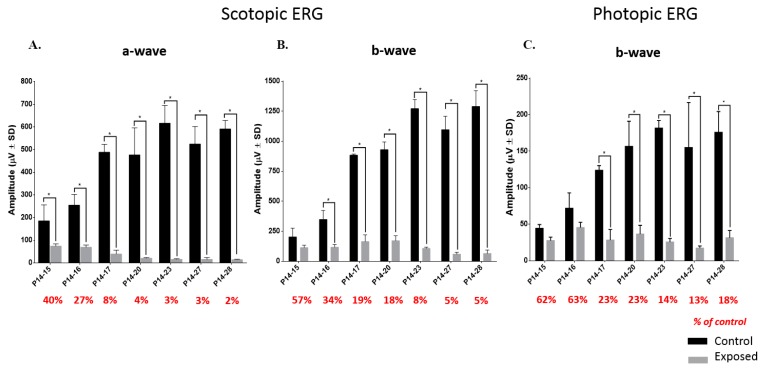
Graphic representation of the global retinal function [(**A**) scotopic a-wave; (**B**) scotopic b-wave and (**C**) photopic b-wave] recorded from light-exposed (10,000 lux; gray bars) animals following different exposure regimens and from age-matched control (unexposed; black bars) groups (*n* = 3–4 animals per group). Asterisks represent statistically significant differences (* *p* < 0.05) compared to age-matched control groups. For each exposed group, the percentage of control is shown below each graph. Amplitudes are reported as mean ± 1SD.

In control animals aged P15, the scotopic recordings (Figure 1 and Figure 2) are mostly composed of an a-wave and with time there is a gradual increase in amplitude of both a- and b-waves to reach a maximal amplitude at age P23. A similar maturation sequence was also observed for the photopic b-wave with a maximum also reached at age P23, after which retinal function remained relatively constant until P28 (Figure 1 and Figure 2). In exposed animals, a significant decrease of all ERG components was already evidenced following 1 day of exposure (Figure 1; P14–15 responses) and with increasing duration of exposure, the a- and b-waves were differently affected. While the a-wave gradually decreased in amplitude (which was more pronounced within the first 3 days of exposures), the b-wave (scotopic and photopic) showed a transient growth in amplitude (between P15 and P20) that was followed by a gradual decrease with longer exposures (also shown in group data at Figure 2). However, comparison with age-matched controls revealed that the loss of function was most pronounced within the first 3 days of light exposure, after which, a plateau-like effect was noted (as per % of control values in Figure 2). Statistical analysis revealed that the scotopic a-wave was significantly different from controls after 1 day of exposure [(at P15), *p* = 0.05, unpaired *t*-test], the scotopic b-wave after 2 days of exposure [(at P16), *p* = 0.004, unpaired *t*-test] and the photopic b-wave after 3 days of exposure [(at P17), *p* = 0.0005, unpaired *t*-test]; a statistically significant difference that remained with longer exposures (Figure 2). 

**Figure 3:**
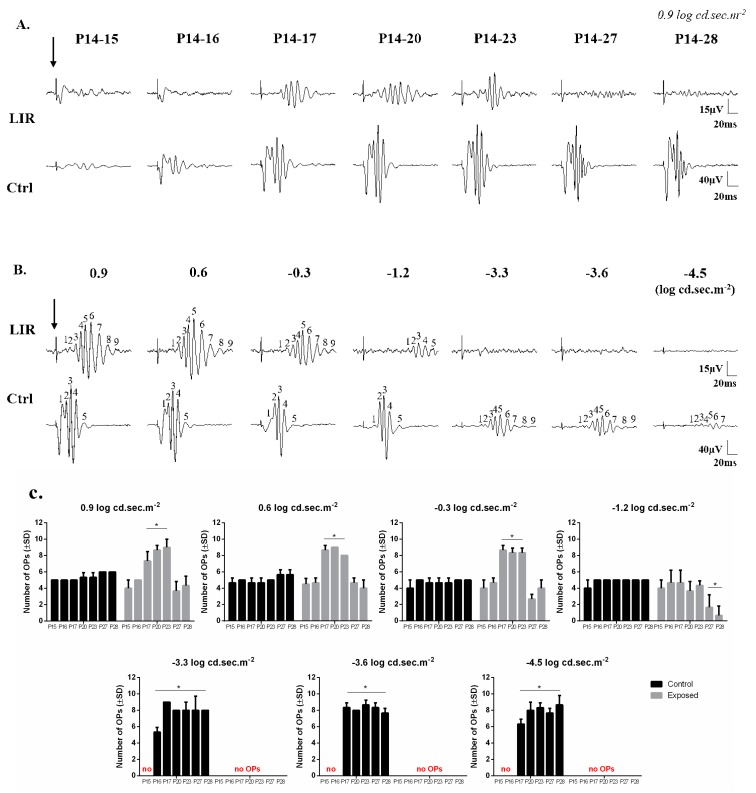
(**A**): Representative scotopic oscillatory potentials (OPs) recorded from light-exposed (10,000 lux; upper tracings) animals following different light exposure regimens and from age-matched control (unexposed; lower tracings) groups (*n* = 3-4 animals per group). Light intensity used: 0.9 log cd.sec.m^−2^. (**B**): Representative scotopic oscillatory potentials (OPs) tracings recorded to stimuli ranging between 0.9 and −4.5 log cd.sec.m^−2^ (as indicated on top of each tracing) from a representative P14–20 LIR animal (upper tracings) and an age-matched control animal (lower tracings). Numbers 1 to 9 indicate the total number of OPs at each intensity in both groups. Horizontal calibration: 20 ms; vertical calibration: 15 µV (exposed) or 40 µV (control). A 20 ms prestimulus baseline is included in all tracings. Vertical arrow indicates the flash onset. Abbreviations: control (Ctrl) and light-induced retinopathy (LIR) animals. OPs recording parameters: bandwidth 100-1000Hz, flash duration 20 µs, inter stimulus interval 10 ms. (**C**): Graphic representation of the number of OPs evoked to different light intensities ranging between 0.9 and −4.5 log cd.sec.m^−2^ (as indicated on top of each bar graph) in control animals (black bars) and following different light exposure regimens (grey bars). Asterisks represent statistically significant differences (* *p* < 0.05) compared to age-matched control groups. Values are reported as mean ± 1SD.

Interestingly, some scotopic ERG tracings evoked to the brightest intensity used (0.9 log cd.sec.m^−2^) appeared to disclose relatively better preserved oscillatory potentials, especially in the P14–17 and P14–20 rats (Figure 1 and Figure 3A). This is best illustrated at Figure 3B with filtered OP tracings evoked to stimuli ranging between (0.9 to −4.5 log cd.sec.m^−2^) and belonging to a representative P14-20 LIR rat (upper tracings) and an age-matched control rat (lower tracings). In control rats (Figure 3B,C), the largest number of OPs (7–9 OPs) was reached in responses evoked to stimuli ranging between −3.3 and −4.5 log cd.sec.m^−2^ in intensity, while brighter (−1.2 to 0.9 log cd.sec.m^−2^) intensities yielded a lower OP count (i.e., 4–6 OPs). In contrast, at high intensities (i.e., between 0.9 and −0.3 log cd.sec.m^−2^), the total number of OP obtained from P14-20 LIR rats was identical to that measured in control rats at dimmer intensities (i.e., between −3.3 and −3.6 log cd.sec.m^−2^). Of interest, the OP sequence appeared to be delayed compared to control (Figure 3). With shorter (P14–15 and P14–16) and longer (P14–27 and P14–28) exposures (Figure 3A), the OPs were almost completely abolished in exposed rats, while they were still present in age-matched control responses.

### 2.2. Effect of Bright Light Exposure on the Retinal Structure

**Figure 4:**
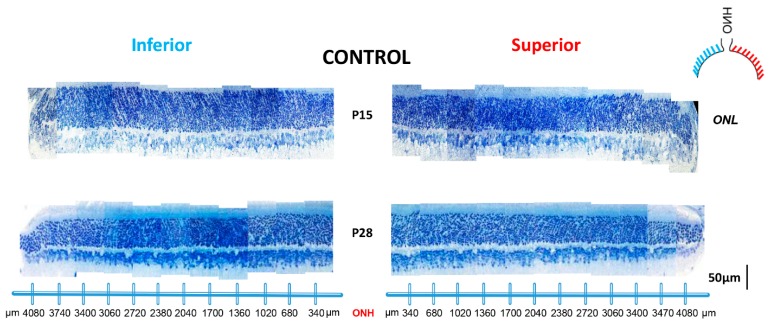
Representative reconstructions of the ONL of the inferior (left) and superior (right) retina (composed of 12–13 consecutive histological segments of 75 µm in width, each sectioned at every 340 µm from the ONH to the ora serrate for each hemiretina) obtained from control animals at P15 and P28. Abbreviations: ONL: outer nuclear layer; ONH: optic nerve head.

**Figure 5:**
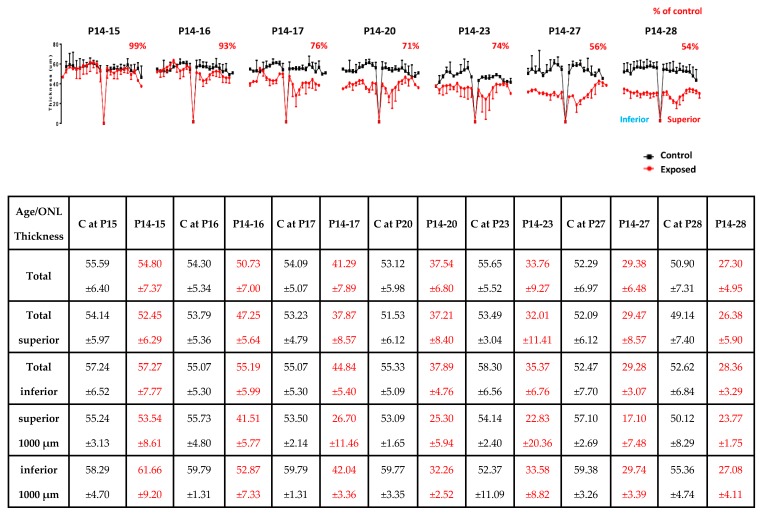
Spidergraph representation of the measurements of ONL thinning (immediately following bright light exposure) across the infero-superior axis in light exposed rats following different light exposure regimens (red) and in age-matched control (black) rats (*n* = 3–4 animals per group). ONL values were taken at every 340 µm from the optic nerve head to the ora serrata in both inferior (left) and superior (right) hemiretinas. Abbreviations: optic nerve head (ONH); control (C). The total ONL loss is indicated in percentage of control for each group above each spidergraph. Group data comparing the ONL thinning (total ONL, superior ONL and inferior ONL loss) between control (black) and exposed animals (red) after different light exposure regimens. Values are reported in mean ± 1SD.

**Figure 6:**
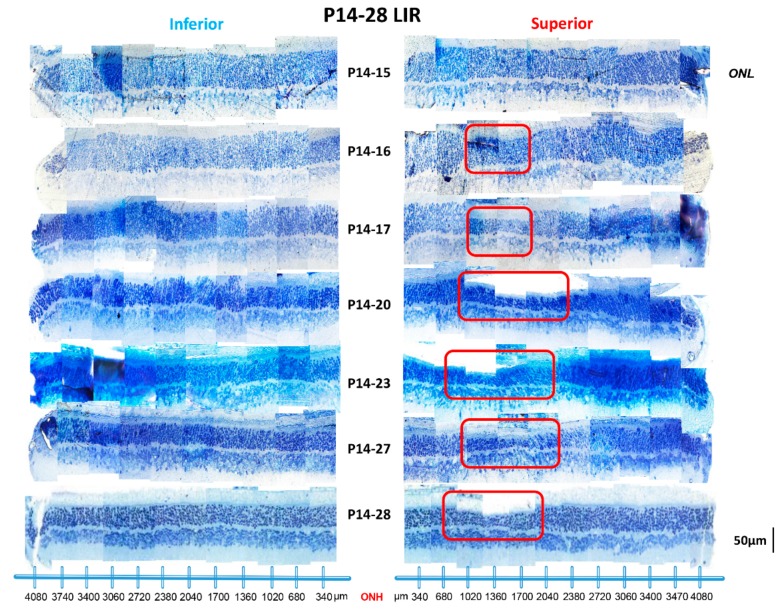
Representative reconstructions of the ONL of the inferior (left) and superior (right) retina (composed of 12–13 consecutive histological segments of 75 µm in width, each sectioned at every 340 µm from the ONH to the ora serrate for each hemiretina) obtained from juvenile exposed animals following different light exposure regimens. Abbreviations: ONL: outer nuclear layer; ONH: optic nerve head. The size of the photoreceptor hole in the superior retina is highlighted with red boxes.

As showed in Figure 4, no significant hemiretinal [total superior retina/inferior retina (SR/IR) ONL ratio: 0.95 and 0.93 at P15 and P28, respectively, *p* > 0.05; Figure 5] or age-dependent differences [total ONL thickness: 55.6 ± 6.40 µm and 50.90 ± 7.31 µm at P15 and P28, respectively (*p* = 0.13, unpaired *t*-test); see table at bottom of Figure 5] in ONL thickness were noticed in normal rats nor after a one-day exposure [total ONL thickness: 99% of control, *p* = 0.05, unpaired *t*-test (Figure 5 and Figure 6)]. A small photoreceptor hole-like area in the superior retina which extended between 680 µm and 1360 µm away from the ONH was created only after 2 days of light exposure (Figure 6). After 3 days of exposure, while the photoreceptor hole did not change, the ONL became significantly thinner within this region [41.51 ± 5.77 µm at P14−16 compare to 26.70 ± 11.46 µm at P14−17 (*p* = 0.03, unpaired *t*-test); Figure 5 and Figure 6]. By P20 (i.e., following P14-20 exposure), the size of this photoreceptor hole had expanded with its boundaries now extending between 680 µm and 2040 µm from ONH (Figure 5 and Figure 6). With longer exposure durations, no further increase in size of the photoreceptor hole could be evidenced (Figure 5 and Figure 6). 

**Figure 7:**
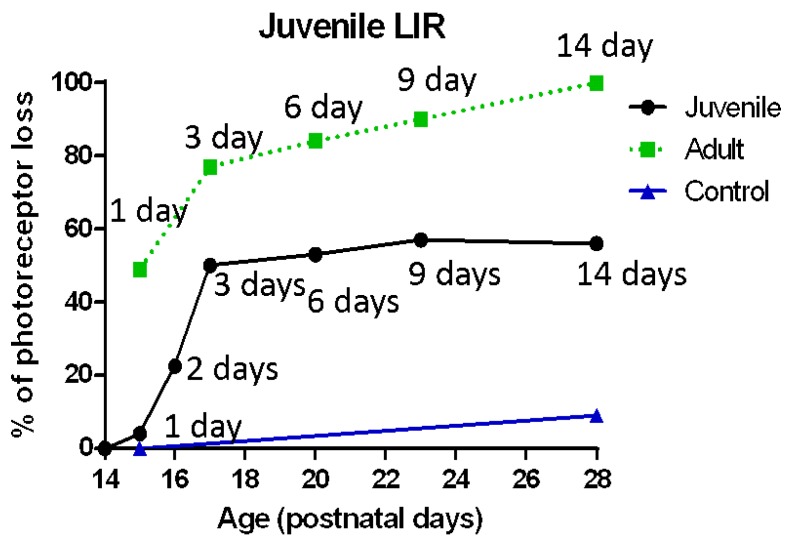
Estimation of photoreceptor loss per day in juvenile (black; *n* = 3–4 animals per group) and adult (green; *n* = 3–9 animals per group) LIR rats compared to control (blue; *n* = 3–4 animals per group) rats. Adult data was taken from Joly et al., 2006 [4] for comparison purposes.

The kinetics of this light-induced ONL thinning are better illustrated at Figure 7, where the amount of photoreceptor loss is plotted against the age at which the light exposure ended in juvenile rats or the duration of exposure (in days) in adult rats (data from previous studies of ours [4]). Our results revealed that in juvenile LIR, the photoreceptor loss peaked after 3 days of exposure following which the photoreceptor degenerated at a much slower rate (% of ONL reduction per day: 1.4% (P14–15), 5.2% (P15–16), 17.1% (P16–17), 5.6% (P17–20 or 1.9% per day), 7.0% (P20–23 or 2.3% per day), 7.5% (P23–27 or 2.5% per day) and 0% (P27–28) (Figure 7). These results showed that the loss of photoreceptors measured after the first 3 days of light exposure was nearly identical to that measured following the next 11 days (23.7% vs 20.1%, respectively; *p* > 0.05, unpaired *t*-test). This contrasted significantly with what was observed in adult rats, where the loss of photoreceptors appeared to be dose-dependent (Figure 7). 

### 2.3. Immunohistochemistry: Juvenile vs. Adults

To better understand the difference in bright light susceptibility of the juvenile and adult retinas, selected cellular and immune responses were assessed and compared between the two groups. A detailed list of the molecular markers used herein is presented in Table 1. 

**Figure 8:**
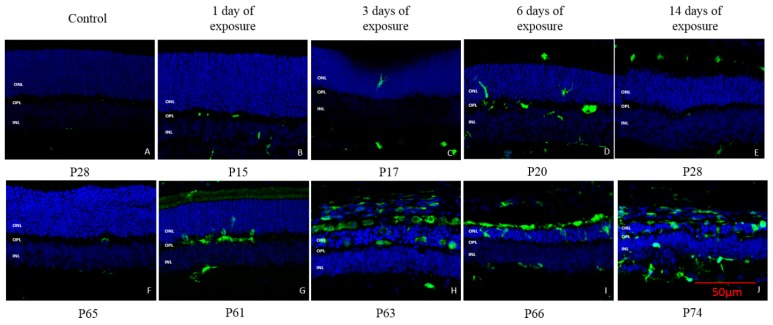
Representative IBA1 staining of microglial cells at each time point of light exposure in the retina of juvenile and adult LIR rats. Green: IBA1 stating. Blue: DAPI. **A**: control juvenile rat at P28; **B**: juvenile rat at P15 after 1 day of light exposure; **C**: juvenile rat at P17 after 3 day of light exposure; **D**: juvenile rat at P20 after 6 day of light exposure; **E**: juvenile rat at P28 after 14 day of light exposure; **F**: control adult rat at P65; **G**: adult rat at P61 after 1 day of light exposure; **H**: adult rat at P63 after 3 day of light exposure; **I**: adult rat at P66 after 6 day of light exposure; **J**: adult rat at P74 after 14 day of light exposure. Calibration bar: 50 µm. Immunohistological observations were based on 3-4 animals per group.

**Figure 9:**
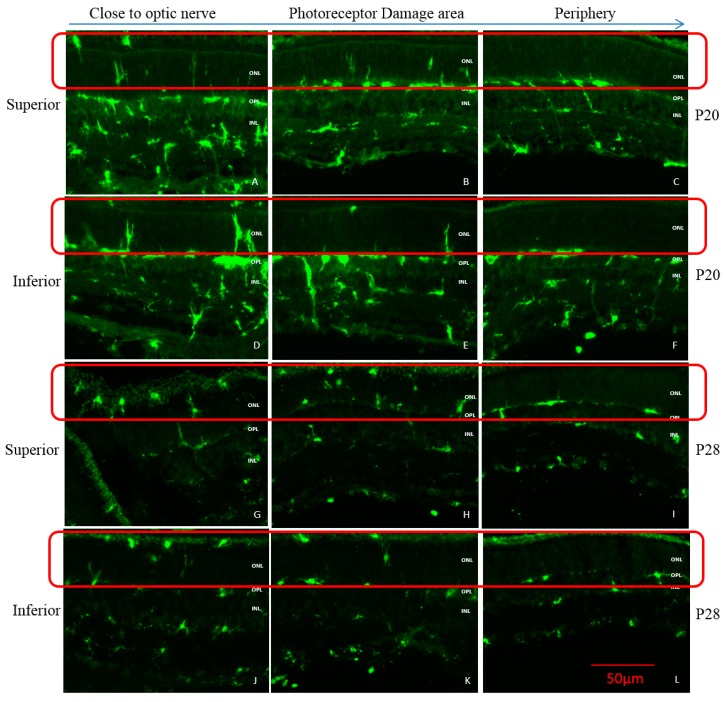
Representative regionalized IBA1 staining of microglial cells (Green) following light exposure in retina of juvenile LIR rats at P20 and P28. Retinal samples were collected at the region close to the optic nerve (**A**,**D**,**G**,**J**), at the photoreceptor damage are (**B**,**E**,**H**,**K**) and at the periphery (**C**,**F**,**I**,**L**) in the superior (A,B,C,G,H,I) and inferior (D,E,F,J,K,L) retinas at P20 (A to F) and P28 (G to L). Red frame: area of subretinal region and ONL showing migration of microglial cells. Calibration bar: 50 µm. Immunohistological observations were based on 3-4 animals per group.

The expression of Iba1 (a macrophage/microglial-specific protein marker) revealed that following light exposure, microglia [usually localized in the OPL and inner retina (INL and IPL)] gradually migrated from the inner retina to reach the outer nuclear layer (ONL) and sub-retinal space in both groups (Figure 8). However, a different pattern of invasion was observed in juvenile retinae compared to adult retinae. In juvenile rats, the first signs of microglial cell migration only appeared after 3 days of light exposure, while in adult rats, 1 day of light exposure was sufficient to trigger this microglial relocation. Moreover, a greater number of microglial cells (i.e., more dense staining) was observed in adult compared to juvenile rats, irrespective of the time point tested, suggesting a stronger microglial activation in the former group (Figure 8). Of note, as shown in Figure 9, more microglial cells were found migrating in the superior retina compared to the inferior retina and these were also found to be more active near the optic nerve head and within the previously reported “photoreceptor hole” area compared to the periphery. 

**Figure 10:**
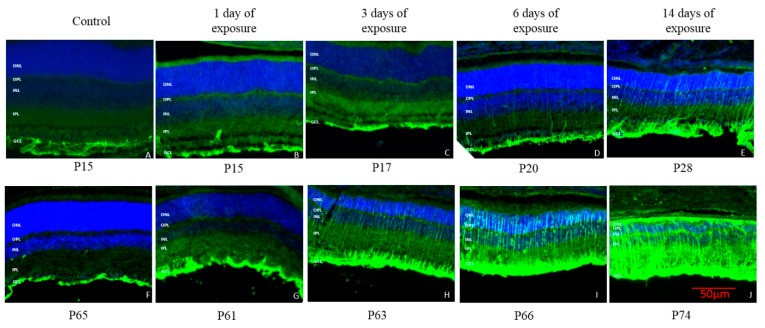
Representative GFAP staining of active Muller cell at each time point of light exposure in retina of juvenile and adult LIR rats. Green: GFAP stating. Blue: DAPI. **A**: control juvenile rat at P15; **B**: juvenile rat at P15 after 1 day of light exposure; **C**: juvenile rat at P17 after 3 day of light exposure; **D**: juvenile rat at P20 after 6 day of light exposure; **E**: juvenile rat at P28 after 14 day of light exposure; **F**: control adult rat at P65; **G**: adult rat at P61 after 1 day of light exposure; **H**: adult rat at P63 after 3 day of light exposure; **I**: adult rat at P66 after 6 day of light exposure; **J**: adult rat at P74 after 14 day of light exposure. Calibration bar: 50 µm. Immunohistological observations were based on 3-4 animals per group.

Müller cell immunoreactivity assessed with GFAP staining, a marker for reactive Müller cells and astrocytes, revealed that the activation of Müller cells began after 3 days of light exposure in both juvenile and adult rats and then gradually increased with the duration of light exposure (Figure 10). However, similar to the Iba1 staining, the expression of GFAP was significantly greater in adult rats compared to juvenile rats, irrespective of the time point tested (Figure 10). 

**Figure 11:**
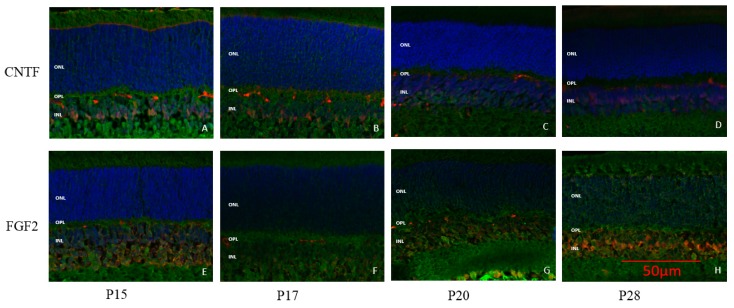
Representative CNTF and FGF2 staining at selected time point in juvenile control retinas. CNTF (**A**,**B**,**C**,**D**) and FGF2 (**E**,**F**,**G**,**H**) staining (in red) are restricted to the INL. A and E: P15; B and F: P17; C and G: P20. D and H: P28. Blue: DAPI. The red spots or lines found in the OPL are non-specific staining bringing by secondary antibody which binds to blood vessels. Calibration bar: 50 µm. Immunohistological observations were based on 3-4 animals per group.

**Figure 12:**
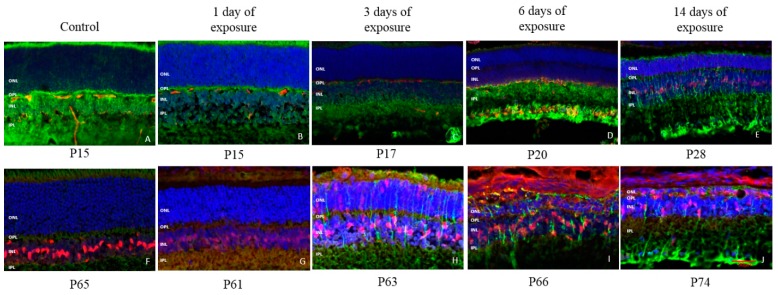
Representative FGF2 and GFAP double staining at each time point of light exposure in retina of juvenile and adult LIR rats. Red: FGF2 staining. Green: GFAP stating. Blue: DAPI. **A**: control juvenile rat at P15; **B**: juvenile rat at P15 after 1 day of light exposure; **C**: juvenile rat at P17 after 3 day of light exposure; **D**: juvenile rat at P20 after 6 day of light exposure; **E**: juvenile rat at P28 after 14 day of light exposure; **F**: control adult rat at P65; **G**: adult rat at P61 after 1 day of light exposure; **H**: adult rat at P63 after 3 day of light exposure; **I**: adult rat at P66 after 6 day of light exposure; **J**: adult rat at P74 after 14 day of light exposure. The red spots or lines found in the OPL are non-specific staining bringing by secondary antibody which binds to blood vessels. Calibration bar: 50 µm. Immunohistological observations were based on 3-4 animals per group.

**Figure 13:**
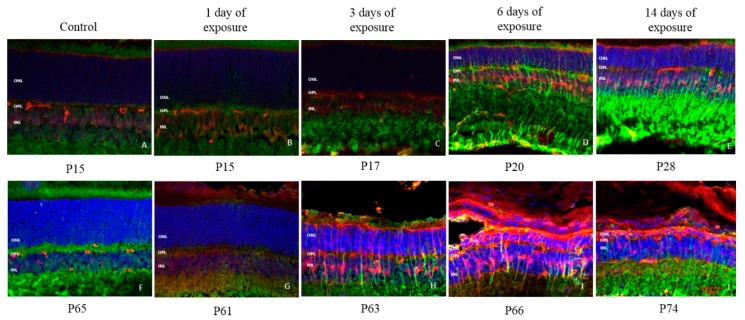
Representative CNTF and GFAP double staining at each time point of light exposure in retina of juvenile and adult LIR rats. Red: CNTF staining. Green: GFAP stating. Blue: DAPI. **A**: control juvenile rat at P15; **B**: juvenile rat at P15 after 1 day of light exposure; **C**: juvenile rat at P17 after 3 day of light exposure; **D**: juvenile rat at P20 after 6 day of light exposure; **E**: juvenile rat at P28 after 14 day of light exposure; **F**: control adult rat at P65; **G**: adult rat at P61 after 1 day of light exposure; **H**: adult rat at P63 after 3 day of light exposure; **I**: adult rat at P66 after 6 day of light exposure; **J**: adult rat at P74 after 14 day of light exposure. The red spots or lines found in the OPL are non-specific staining generated by secondary antibody which binds to blood vessels. Calibration bar: 50 µm. Immunohistological observations were based on 3-4 animals per group.

The neuroprotective effect of two different neurotrophic factors (FGF2 and CNTF) was also examined, results of which are presented in control (Figure 11) and in light exposed rats (Figure 12 and Figure 13, respectively). In juvenile control rats, FGF2 expression was evidenced only at P28 (Figure 11H) and was confined to the INL. In juvenile LIR rats, FGF2 staining was first visible after 14 days of light exposure (i.e., at P28) and was slightly more expressed in the INL (Figure 12E) than in age-matched control groups (Figure 11H). The expression of FGF2 slightly increased with age and also remained within the inner retina in older control animals (as assessed at P65; Figure 12F). In adult exposed rats, FGF2 expression in the ONL was first noticed after 3 days of light exposure (Figure 12H), an upregulation that was preserved even after 14 days of light exposure (Figure 12J). CNTF expression, although weak, was first seen at P15 in control juvenile rats and was also localized to the INL only (Figure 11A). Similar to what was observed with the FGF2 neurotrophic factor, CNTF expression also remained weakly upregulated in the INL until adulthood (i.e., P65; Figure 13F). The intensity of the CNTF staining in juvenile rats increased after 6 days of light exposure (Figure 13D), and even more after 14 days (Figure 13E), and became widespread throughout the inner and outer retinas. In adult rats, 3 days of light exposure (Figure 13H) was sufficient to induce a comparable CNTF reaction as that observed after a 14 days long exposure period in juvenile rats (Figure 13E).

## 3. Discussion

### 3.1. Electrophysiological Findings

Electrophysiological findings obtained immediately following the cessation of light exposure (the acute phase) in juvenile LIR suggest a dose-independent effect, as damage was most pronounced within the first three days of exposure, after which a plateau-like effect was observed (Figure 2 and Figure 6). Furthermore, scotopic ERGs were more attenuated than photopic ones, findings in accord with previous studies that showed that LIR is a rhodopsin-mediated retinopathy [3,4,6,14]. Interestingly, while a decrease in amplitude was observed for the a-wave, a small but transient increase of the b-wave was noted between P15 and P20 (for exposures between P14–15 and P14–20). Since the retina is exposed to light during a time period where it is known to mature [15,16], a synaptic reorganization (between the photoreceptor and the cells of the inner retina) could have taken place in order to compensate for this initial loss of the photoreceptor signal. Of note, Polosa et al. 2016 [6] showed a relative preservation of the visual evoked potential (VEP) despite a significant attenuation of fERGs, findings in support of the latter claim. Unfortunately, previous results of ours also suggest that the latter synaptic reorganization cannot continue to compensate for the progressive loss of photoreceptors that continues long after the cessation of light-exposure [4,6,17,18].

Of interest, it is following the P14–P20 exposure that the b-wave reached maximal amplitude (Figure 1 and Figure 2) and where the OPs were most prominent (Figure 3). The OPs are believed to reflect the synaptic activity of inhibitory feedback processes generated mainly by the amacrine cells, although contributions of bipolar cells and other inner retinal cells are not completely excluded [19,20,21]. Horsburgh and Sefton (1987) [22] showed that in the developing rat retina, the synaptogenesis of amacrine and bipolar cells begins at around P11 to P13 and increases until adulthood. As both cell types are said to participate in the genesis of the b-wave and the OPs, our findings would also suggest that bright light exposure does not affect the maturation of the inner retina, as a growth of the b-wave and prominence of OPs were evidenced despite a gradual decrease of the a-wave, at least with the shorter exposures. Of note, the OP waveforms of LIR rats evoked to the highest intensity (0.9 log cd.sec.m^−2^) are identical to those of unexposed animals evoked to lower intensities (−3.3 to −3.9 log cd.sec.m^−2^) (Figure 1 and Figure 3B), confirming the lost in phototransduction capability of the LIR rat photoreceptors resulting from the shortening of the outer segments caused by the bright light exposure [6] and ensuing inner retinal activation.

### 3.2. Histological Findings

Histological findings correlated with ERG findings in showing a greater loss of photoreceptors with the first 3 days of exposure (Figure 2 and Figure 5). Furthermore, in the acute phase, a minimum of 2 days of exposure was required to produce retinal damage with the typical hemiretinal disparity (i.e., formation of the photoreceptor hole in the superior retina) (Figure 5 and Figure 6) that characterizes the rodent model of LIR [4,5,6,23,24]. In addition, while the size of the photoreceptor hole grew between P16 and P20, it only progressed towards the ora serrata side [i.e., from 680 (temporal to the ONH) to 1360 µm (at P15); from 680 to 1700 µm (at P28)]. The higher resistance of the photoreceptors next to the ONH is interesting and remains to be elucidated. To our surprise, with longer exposures, no further increase in size of the photoreceptor hole was observed such that the dimension of the photoreceptor hole obtained following a 6 day exposure was equivalent to that measured following a 14 day exposure.

Why is it that, in juvenile rats, exposures longer than 3 days do not result in a greater loss of photoreceptor cells? It could be that the retina of juvenile rats continues to mature despite the bright light exposure and, consequently, the photoreceptor cells that were normally programmed to die (i.e., apoptosis) replaced those that died as a result of bright light exposure. Consequently, preservation of these cells could have resulted in limiting retinal damage, a retinal plasticity that is no longer possible in older animals. As shown at Figure 7, we estimated (from previous work [4]) that this ‘‘maturational’’ apoptosis would account for a loss of approximately 9% in retinal thickness.

### 3.3. Immunohistochemisty Findings

The data obtained at IHC suggests fundamental differences between the adult and juvenile retina that could explain their different reaction to bright light exposure. In juvenile rats, three days of light exposure were necessary to trigger a measurable inflammatory response compared to only one day in adult rats. In addition, at each time point, juvenile rats showed a less intense microglial migration than adult rats (Figure 8), suggesting that juvenile rats had a delayed and weaker inflammatory response compared to adult rats after being exposed to light. Given that in LIR, microglial activation in the retina is triggered by the degenerating photoreceptors [25], it is reasonable to assume that juvenile rats had less photoreceptor damage than adult rats. This conclusion is further supported by the fact that microglia was more activated in the superior retina after light exposure, that is the retinal region previously shown to yield the most important light-induced degeneration (Figure 9) as previously reported by us and others [3,4,5,6]. Furthermore, it was shown that in LIR, the microglia can either act as a destructive pro-inflammatory polarization or a protective anti-inflammatory polarization [26]. Given that in juvenile rats there is no further damage following the onset of the inflammatory response, this would suggest a protective anti-inflammatory response. In contrast, results obtained in adults either suggest a destructive pro-inflammatory reaction or that the light damage was simply too severe for the immune reaction to deal with it efficiently. The latter would therefore point to the significant role played by the immune system in causing the severe retinal degeneration that continues to progress long after the cessation of light exposure, a claim also advanced to explain the pathophysiology of other retinal degenerative disorders such as Age-related Macular Degeneration, Diabetic Retinopathy and Retinitis Pigmentosa [27,28].

Müller cells were found to be activated in LIR simultaneously with an increased expression of CNTF and FGF2 in the outer retina, thus confirming previous findings of ours which showed that Müller cells actively upregulated the expression of neurotrophic factors in this layer [7]. These neurotrophic factors were expressed later (after 6 days for CNTF and after 14 days of exposure for FGF2) in juvenile rats compared to adult rats (after 3 days of exposure for CNTF and FGF2), their expressions increasing with longer exposures. The weaker and delayed upregulation of neurotrophic factors in very young retinas correlates well with the late activation of microglial cells, which was first observed after 3 days of exposure. This delayed immune response could also explain why most of the damage (as assessed with fERG and retinal histology) occurred within the first 3 days of light exposure in young animals (Figure 7). In contrast, the later upregulation of neurotrophic factors in juvenile retinas correspond with a reduced structural and functional damage, findings highlighting once again the neuroprotective effect of these factors against light-induced oxidative stress. Of note, our results also show that in LIR, the immune response is triggered prior to the expression of neurotrophic factors, findings that are in accord with other studies [9]. In adult rats, although the expression of neurotrophic factors was observed earlier and followed a dose-dependent manner (i.e., stronger staining with longer exposures), the combination of light exposure with the immediate and potent response of the microglia (already seen after 1 day of light exposure) most probably enhanced the effect of the ensuing oxidative stress to the point that it became too difficult for the inherent retinal defense mechanisms (i.e., immune system/neurotrophic factors) to efficiently eliminate it over time.

## 4. Materials and Methods

All experiments were performed in compliance with the Association for Research in Vision and Ophthalmology (ARVO) Statement for the use of animals in ophthalmic and vision research as well as with the guidelines of the Canadian Council on Animal Care (CCAC: http://www.ccac.ca) and were approved by the McGill University-Montreal Children’s Hospital animal care committee (AUP2001-4164), an Institutional Review Board accredited by the CCAC.

### 4.1. Light Exposure Procedure

Light exposure protocol was performed as previously reported [6,17,18]. At postnatal (P) day 14 (which corresponds to eye opening in rats), four litters (*n* = 48) of Sprague Dawley (SD) albino rats (Charles River Laboratories, St-Constant, QC, Canada) were exposed to a bright cyclic light (10,000 lux, 12 h dark/12 h light) for 1 (P14–P15), 2 (P14–P16), 3 (P14–P17), 6 (P14–P20), 9 (P14–P23), 13 (P14–P27) and 14 (P14–P28) consecutive days (d). Mothers were excluded from this study. Similarly, adult rats (*n* = 24) were also exposed to the same light regimen for 1 (P60–61), 3 (P60–63), 6 (P60–66) and 14 (P60–P74) consecutive days. Aged-matched pup (*n* = 48) and adult (*n* = 24) rats served as control. Prior to testing, all control animals were raised in the normal light environment (80lux, 12h dark/12h light) of the animal facility. All post-light exposure tests were performed immediately following the cessation of the bright light exposure regimen (*n* = 3–4 per group). Please note that since the effect of different light exposure durations was previously well described for adult rats at the retinal function (fERG) and structural (retinal histology) level [4,6,7], only newly acquired data for adult rats is reported in this study (i.e., immunohistological experiments only).

### 4.2. ERG Recordings

Scotopic and photopic flash ERGs were recorded following an overnight dark adaptation period of 4 h and analyzed as previously described [6,17,18]. Briefly, ERGs were recorded with a DTL fiber electrode (27/7 X-Static silver coated conductive nylon yarn, Sauquoit Industries, Scranton, PA, USA) that was placed centrally on the cornea and held in place with a moisturizing solution (Tear-Gel, Novartis Ophthalmic, Novartis Pharmaceuticals Inc, Dorval, Quebec, Canada). The reference (GrassE5 disc electrode) and ground (Grass E2 subdermal electrode) electrodes were positioned in the mouth and tail, respectively. Recordings of full-field ERGs (bandwidth: 1–1000Hz; 10000×; 6db attenuation; GrassP-511 amplifiers) and OPs (bandwidth: 100-1000Hz; 50000×; 6db attenuation; GrassP-511 amplifiers) were performed simultaneously with the Biopac data acquisition system (Biopac MP100WS, Biopac System Inc., Goleta, CA, USA). Scotopic ERGs were obtained in response to progressively brighter flashes of white light ranging in intensity from −6.3 log cd.sec.m^−2^ to 0.9 log cd.sec.m^−2^ in 0.3 log-unit increments [Grass PS-22 photostimulator, inter stimulus interval: 10 s, flash duration 20 μs, average of 3–5 flashes depending on intensity]. Photopic ERGs were evoked to flashes of 0.9 log cd.sec.m^−2^ (photopic background: 30 cd/m^2^, inter stimulus interval: 1sec, flash duration 20 μs, average of 20 flashes). In order to avoid the previously reported light adaptation effect, the photopic recordings were obtained 20 min following the opening of the background light.

Given the young age of animals and the feeding requirements, animals were dark adapted for 4 h only, a duration that was previously shown to yield, in SD rats, a nearly normal scotopic retinal sensitivity as per ERG [29]. The amplitudes of the a- and b- waves of the scotopic mixed rod-cone response (ERG response evoked to the brightest flash delivered in scotopic conditions) and the photopic b-waves are reported in this study.

### 4.3. Retinal Histology

The retinal histology was performed according to our previous reports [4,6,17,18]. Retinal images were taken with the AxioVision 4.8.2.0 software (Carl Zeiss Microscopy GmbH, Jena, Germany). Twelve consecutive histological segments of 75 µm in width, each sectioned at every 340 µm from the ONH to the ora serrata were taken from the superior and inferior retinas to evaluate the extent of retinal damage. The thickness of the outer retinal layer (ONL) of each segment is presented in spidergraph forms. The dimension of the photoreceptor hole was determined as the area comprised between the region of maximal damage (i.e., thinnest ONL) to the retinal position (towards the ONH or ora serrata) where the ONL had returned to a constant [variation of ±1 row] and uniform thickness, as previously demonstrated [6,17].

### 4.4. Immunohistochemistry

Eyes were collected as previously described [30] at selected time points after light exposure cessation (P15, P17, P20 and P28 for juvenile; P61, P63, P66 and P74 for adults). Primary and secondary antibody concentrations are showed in Table 1. Retinal images were taken with a fluorescence microscope (Leica DMI6000) or a confocal microscope (Zeiss LSM 780). Controls included negative control (unexposed juvenile and adult age-matched rats) and blank controls (staining with primary antibodies omitted) to control for the non-specific staining. The reagents used are also listed in Table 1.

### 4.5. Statistical Analysis

Statistical significance was determined using a Student’s *t*-test (Prism 6.0 software; Graph Pad, San Diego, CA, USA). All values are reported as mean ± 1 standard deviation (SD).

## Figures and Tables

**Table 1:** Immunohistochemistry reagents.

	Molecular Markers	Source	RRID	Dilution	Antibody
Primary	IBA1	Wako Chemicals 019-19741	AB_839504	1:500	Rabbit Polyclonal
	GFAP	Millipore sigma G3893	AB_477010	1:100	Mouse Monoclonal
	FGF2	Millipore sigma 05-118	AB_309633	1:100	Mouse Monoclonal
	CNTF	Millipore sigma MAB338	AB_2083064	1:100	Mouse Monoclonal
Secondary	Alexa Fluor® 488	Abcam ab150077	AB_2630356	1:1000	Goat anti rabbit IgG
	Alexa Fluor® 594	Abcam ab150116	AB_2650601	1:1000	Goat anti mouse IgG
Mount medium	ProLong®Gold Antifade Mountant with DAPI	Thermo fisher scientific P36935	N/A	N/A	N/A

## Abbreviations

LIR	Light-induced retinopathy
ERG	Electroretinogram
OPs	Oscillatory Potentials
IHC	Immunohistochemistry
GFAP	linear dichroism
CNTF	Cilliary neurotrophic factor
FGF2	Fibroblast growth factor 2
IBA1	Ionized calcium binding adaptor molecule 1
DAPI	4′,6′-diamidino-2-phenylindole
ONL	Outer nuclear layer
OPL	Outer plexiform layer
INL	Inner nuclear layer
ONH	Optic nerve head
SR	Superior retina
IR	Inferior retina
